# Importance of xenarthrans in the eco-epidemiology of *Paracoccidioides brasiliensis*

**DOI:** 10.1186/1756-0500-2-228

**Published:** 2009-11-17

**Authors:** Virgínia B Richini-Pereira, Sandra MG Bosco, Raquel C Theodoro, Lígia Barrozo, Silvia CB Pedrini, Patrícia S Rosa, Eduardo Bagagli

**Affiliations:** 1 Departamento de Microbiologia e Imunologia, Instituto de Biociências de Botucatu, Universidade Estadual Paulista, Botucatu, SP, Brasil; 2 Departamento de Geografia, Faculdade de Filosofia, Letras e Ciências Humanas, Universidade de São Paulo, São Paulo, SP, Brasil; 3 Instituto Lauro de Souza Lima, Bauru, SP, Brasil

## Abstract

**Background:**

Several pathogens that cause important zoonotic diseases have been frequently associated with armadillos and other xenarthrans. This mammal group typically has evolved on the South American continent and many of its extant species are seriously threatened with extinction. Natural infection of armadillos with *Paracoccidioides brasiliensis *in hyperendemic areas has provided a valuable opportunity for understanding the role of this mammal in the eco-epidemiology of Paracoccidioidomycosis (PCM), one of the most important systemic mycoses in Latin America.

**Findings:**

This study aimed to detect *P. brasiliensis *in different xenarthran species (*Dasypus novemcinctus*, *Cabassous *spp., *Euphractus sexcinctus*, *Tamandua tetradactyla *and *Myrmecophaga tridactyla*), by molecular and mycological approaches, in samples obtained by one of the following strategies: i) from road-killed animals (n = 6); ii) from naturally dead animals (n = 8); iii) from animals that died in captivity (n = 9); and iv) from living animals captured from the wild (n = 2). Specific *P. brasiliensis *DNA was detected in several organs among 7/20 nine-banded armadillos (*D. novemcinctus*) and in 2/2 anteaters (*M. tridactyla)*. The fungus was also cultured in tissue samples from one of two armadillos captured from the wild.

**Conclusion:**

Members of the Xenarthra Order, especially armadillos, have some characteristics, including a weak cellular immune response and low body temperature, which make them suitable models for studying host-pathogen interaction. *P. brasiliensis *infection in wild animals, from PCM endemic areas, may be more common than initially postulated and reinforces the use of these animals as sentinels for the pathogen in the environment.

## Findings

Great advances have been made over the last few years in understanding the evolutionary aspects of fungal groups that attach themselves to animal hosts [1]. Xenarthrans (armadillos, sloths and anteaters) are ancient mammals that appeared in South America 65 million years ago. While many members of xenarthrans became extinct on account of different factors (environmental changes, anthropic actions) and some of them are still considered critically endangered or vulnerable, some other Xenarthran species, such as nine-banded armadillos, are still particularly abundant in the central and southern regions of South America, despite intensive hunting [2,3]. The ancient South American ancestry of xenarthrans, and their terrestrial and arboreal niches, suggest that they may have coexisted in close proximity with several Neotropical pathogens, including onygenalean fungi such as *Paracoccidioides brasiliensis*, *Histoplasma capsulatum *and *Coccidioides immitis *[1].

*Paracoccidioides brasiliensis *is an important pathogenic fungus that causes paracoccidioidomycosis (PCM), a systemic mycosis with broad distribution in Latin America. The infection is caused by inhalation of airborne propagules of the mycelial phase of the fungus that reach the lungs and differentiate into the yeast parasitic phase [4].

The precise habitat of *P. brasiliensis *has remained undefined, even after a century of ongoing discoveries about the disease. The high frequency of isolation or molecular detection of the fungus in armadillos has created new opportunities for the study of the role of xenarthrans in the ecology of this microorganism. The first isolation of *P. brasiliensis *was observed in armadillos from the Amazon region [5], a finding that has been confirmed by several other research groups in Brazil [6-10] and Colombia [11,12].

The present study aimed to evaluate *P. brasiliensis *infection in tissue samples of several xenarthran members, by using molecular and traditional mycological procedures. The data indicate that, besides armadillos, the pathogen may occur in other xenarthran terrestrial species.

### Study area, animals evaluated and sampling procedures

The xenarthran animals (n = 25) were obtained in the Botucatu endemic PCM area, in São Paulo State, Brazil, by one of the following strategies: i) road-killed animals (n = 6), that had been recently killed (1-7 hours) and presented no exposure of the internal organs; ii) naturally-dead armadillos (n = 8), which were found in the field of the Lauro de Souza Lima Institute (ILSL), Bauru County, a center of leprosy study located in a savanna reserve; iii) armadillos that died in captivity (n = 9), after having been maintained in the armadillo facility of ILSL; iv) armadillos captured from the wild (n = 2) in Cerqueira César County, that proved to present one of the highest prevalences for human PCM disease [13,14]. The animals obtained from the ILSL (procedures ii and iii) were maintained frozen for weeks to months until the necropsy and tissue sample collection. All animal tissues were analyzed molecularly while the culture and histopathology were evaluated in the armadillos captured from the wild (procedure iv).

The data on the geographical location of the animals and results are summarized in Table 1.

**Table 1:** Geographic, environmental and sex data from wild animal species evaluated for molecular detection of *P. brasiliensis*.

Animal	characteristics	Species	Sex	Geographic location	Environmental features	Tissue/Nested-PCR (+ or -)
1			NA	23°04'00.58"S 47°36'24.50"W	Ombrophylous forest. soil: ultisol. AR:1280 mm	lu(-), h (-)
2		*D. novemcintus*	male	22°53'09.20"S 48°27.35.59"W		**lu(+), s(+), l(+), k(+)**, h(-), **mln(+)**
3		*E. sexcinctus*	male	22°46'8.09"S 48°33'45.80"W	Contact Savanna/STF. soil: latosol. AR 1360 mm	lu(-), s(-), l(-), mln(-)
4	road-killed	*T. tetradactyla*	male	23°02'38.94"S 48°31'10.59"W	STF. soil: latosol. AR:1400 mm	lu(-), s(-), l(-), h(-), mln(-)
5			male	22°55'57.01"S 48°20'34.05"W	STF. soil: ultisol. AR:1400 mm	**lu(+), s(+)**, l(-), **mln(+)**
6		*M. tridactyla*	female	23°01'51.18"S 48°30'47.26"W	STF. soil: latosol. AR:1400 mm	**lu(+), s(+), l(+)**, k(-), h(-), **mln (+)**, ag(-)

7			female			lu(-), s(-), **l(+)**, mln(-)
8			female			lu(-), **s(+)**, l(-), mln(-)
9			male			lu(-), s(-), l(-), mln(-)
10			female			**lu(+)**, s(-), l(-), mln(-)
11	died naturally		male			lu(-), **s(+), l(+), mln(+)**
12			female			**lu(+), s(+), l(+), mln(+)**
13			male			lu(-), s(-), l(-), mln(-)
14			male	22°19'39.52"S	Savanna. AR 1360 mm.	lu(-), s(-), l(-), mln(-)
					
15		*D. novemcintus*	male	48°57'31.09"W	soil: sandy, latosol, pH 3.8, H+Al 58 mmol_c_/dm^3^.	lu(-), s(-), l(-), mln(-)
16			male	range 2525 m^2^		lu(-), s(-), l(-), mln(-)
17			male			lu(-), s(-), l(-), mln(-)
18			male			lu(-), s(-), l(-), mln(-)
19	died in captivity		male			lu(-), s(-), l(-), mln(-)
20			male			lu(-), s(-), l(-), mln(-)
21			male			s(-), l(-), mln(-)
22			female			s(-), l(-), mln(-)
23		*Cabassous *spp.	female			lu(-), s(-), l(-), mln(-)

24	animals		male	22°55'66"S 49°05'18.7"W	STF. AR:1360 mm. soil: sandy, latosol, pH 5.2, H+Al 25 mmol_c_/dm^3^.	**lu(+), s(+), l(+)**, k(-), **mln (+)**, ag(-)
25	captured from the wild	*D. novemcintus*	male	23°03'21.69"S 49°11'29.41"W	STF. AR:1360 mm soil: clayed, latosol, pH 4.5, H+Al 81 mmol_c_/dm^3^.	lu(-), s(-), l(-), k(-), h(-), mln (-), ag(-)

lu, lung; s, spleen; l, liver; k, kidney; h, heart; mln, mesenteric lymph node; ag, adrenal gland. STF, Semideciduous tropical forest; AR-annual rainfall, NA-not available.

### Molecular detection

The DNA extraction was performed by grinding the liquid-nitrogen frozen tissue sample with mortar and pestle as proposed by Corredor et al. [11]. The molecular detection was carried out by Nested-PCR reactions, using as outer primers the panfungal primers ITS4 (5'-TCCTCCGCTTATTGATATGC-3') and ITS5 (5'-GGAAGTAAAAGTCGTAACAACG-3'), at the annealing temperature of 60°C [15] and inner primers PbITSE (5'-GAGCTTTGACGTCTGAGACC-3') and PbITSR (5'-AAGGGTGTCGATCGAGAGAG-3'), annealed at 62°C [16]. The amplicons were purified by the commercial kit GFX-PCR-DNA and Gel-Band Purification (GE-Healthcare) and the sequencing reactions were carried out on both strands in a MegaBace™ 1000 (GE-Healthcare). The sequences were compared to the NCBI database by using BLASTn (Basic Local Alignment Tool for nucleotide).

Positive amplifications of specific amplicons of *P. brasiliensis *were detected in several tissue fragments of seven *D. novemcinctus *armadillos and two *M. tridactyla *anteaters (Table 1).

Concerning the armadillos from the ILSL, in the savanna reserve, the positivity was high (62.5%) among the ones found dead in the field or those had been recently introduced into captivity, when compared with the ones that had died after a long period in captivity (all animals negative).

When submitted at BLASTn analysis, all the purified and sequenced amplicons showed 99% identity with *P. brasiliensis *sequences deposited at the GenBank (Table 2).

**Table 2:** Identity percentages of amplicons obtained in wild animals positive for *P. brasiliensis *from deposited homologue rDNA sequences, as determined by BLASTn analysis.

animal	source*	species	% identity/GenBank acess
2	i	*D. novemcintus*	99%/AY374336.1
5	i	*M. tridactyla*	99%/AY374336.1
6	i	*M. tridactyla*	99%/AY374336.1
7	ii	*D. novemcintus*	99%/AB30448.1
8	ii	*D. novemcintus*	99%/AB30448.1
10	ii	*D. novemcintus*	99%/AB30448.1
11	ii	*D. novemcintus*	99%/AB30448.1
12	ii	*D. novemcintus*	99%/AB30448.1
24	iv	*D. novemcintus*	99%/AB30448.1

* according to the strategy employed: i) road-killed; ii) died in the wild; iii) died in captivity; iv) free-living animals.

### Fungal culture and histopathological analysis

The captured armadillos were euthanatized and aseptically necropsied; fragments of liver, spleen and mesenteric lymph nodes were cultured on Mycosel™ agar (Becton Dickinson and Company, Cokeysville, MD, USA), supplemented with gentamicin (50 μg/mL) and incubated for 4 weeks at 36°C, as already described [6,7]. The characteristic yeast colony of *P. brasiliensis *was recovered from the spleen and mesenteric lymph nodes of one animal. Histopathological sections stained with hematoxylin-eosin, periodic-acid-Schiff and Gomori-Groccot methanamine silver stain were also carried out and did not reveal the presence of the fungus or granuloma in either animal. Molecular analysis also confirmed the infection of the culture-positive armadillo.

The study was performed in accordance with the Brazilian Agency for Protection of the Environment and Renewable Resources (IBAMA, license number 12408-2) and Animal Research Ethics Committees (CEEA, authorization number 052/05) at the Institute of Biosciences, UNESP-Botucatu.

## Discussion

The present study confirms the frequent occurrence of *P. brasiliensis *in nine-banded armadillos and also provides the first indication that the infection occurs in the anteater *M. tridactyla*, another xenarthran that also has the habit of foraging in soil. In a previous study of road-killed wild animals, it was observed that besides the nine-banded armadillo (*D. novemcinctus*), the seven-banded armadillo (*Dasypus septemcinctus*) also might be infected by the fungus [17]. In a Colombian study, Restrepo's group has isolated *P. brasiliensis *from the naked-tailed armadillo *Cabassous centralis *[12]. As already suggested, *P. brasiliensis *infection in wild animals, from PCM endemic areas, may be more common than initially postulated, especially in armadillos and probably other xenarthrans. These animals have some peculiar physiological and ecological characteristics such as a low body temperature, besides the fact that most of them, such as armadillos, live literally immersed in soil and organic matter, mainly in tropical and subtropical regions, under biotic and abiotic conditions that promote multiple encounters with a diverse group of pathogens and vectors. Furthermore, these animals are thought to possess a weak cellular immune response. At first, this seems to be somewhat controversial since no animal presenting PCM disease has been detected; however, the damage to the host does not depend only on the pathogen, but also on the host response. Casadeval and Pirofisk [18] clarified many points on virulence and pathogenicity regarding host immune response and pathogen activity. According to the authors, there are six classes of pathogenic microorganisms varying from those that cause damage in hosts with an extremely weak immune response to others which cause disease only in a situation of very strong immune response [18]. Therefore, it seems reasonable to consider *P. brasiliensis *to be a pathogen whose ability to provoke disease also depends on host immune response. Since the cellular immune response is weak in armadillos, it is possible to detect yeast cells in many of their organs; however, this is not sufficient to cause disease as observed in human hosts. Taken together, these factors make xenarthrans suitable models for studying host-pathogen interaction [19]. Given that many of the extant xenarthrans species are seriously threatened with extinction, seriously limiting the sampling of free-living animals, the present strategy focusing on road-killed animals and those having died in the wild or having been sacrificed for other studies could represent a viable alternative for studying natural infections in these peculiar mammals.

The molecular approach applied herein is both sensitive and specific, since the inner primers used in the second PCR were designed specifically for *P. brasiliensis *and anneal in the ITS1 region, known to be variable among different species. The specificity of this primer set has been previously verified and no amplification has been detected in several other related species such as *Histoplasma capsulatum*, *Blastomyces dermatitidis*, *Coccidioides *spp. and *Emmonsia parva *[16,17].

In the group of armadillos originally obtained in the savanna reserve at Bauru/SP, the positivity of Nested-PCR appears to show two distinct situations: while the positivity was relatively high in the animals found dead of natural causes in the field and in those that died soon after being introduced into captivity, the molecular detection was negative in all samples from the animals that had been maintained for a long period in captivity. Although keeping the animal corpses frozen until the necropsy may harm the molecular analysis, it could also be argued that during captivity, the animals had become free of the pathogen. Recently, we have also confirmed that the armadillos living in this area are highly infected by the fungus, since *P. brasiliensis *was detected in three of the four captured animals that were evaluated by fungal culture and molecular detection [10]. A survey of nine-banded armadillos captured in Manduri County, also considered a hyperendemic area for human PCM disease, demonstrated 100% positivity for fungal culture [7]. In an attempt to evaluate the PCM disease development in nine-banded armadillos, a group of them were captured in this county and maintained in captivity during a three-year evaluation period. Curiously, none of them presented any clinical evidence of active PCM disease nor was the fungus detected in histopathological sections [20]. These findings support the idea that free-ranging armadillos from an endemic area may be continuously acquiring the fungus through their daily contact with soil and when they are removed from the natural sources of infection, a process of fungal clearance from the animal tissues may occur [19,20].

In the present study, *P.brasiliensis *was also isolated from the spleen and mesenteric lymph node in one of the two armadillos captured in the Cerqueira César County, a location identified as presenting one of the highest prevalences of human PCM cases in our hyperendemic area [13,14,21]. In addition, fungal DNA amplification with specific primer was positive in the same specimen. Histopathological observations of liver, lung and spleen tissue of both of the cultured armadillos did not reveal fungal lesions. These results are in accordance with previous findings which indicate that armadillos do not develop the PCM disease at a high frequency when compared with the high overall infection rates [6,8,20]. On the other hand, the histopathology for detecting the fungus in dermatological lesions of patients with PCM was more sensitive than molecular protocols and culture [22]. A possible explanation for this fact may be the difference in immune response between humans and armadillos. While in humans, well-defined granulomas are more frequent, in armadillos they tend to be more poorly defined [7,9,20].

Since many xenarthrans, especially armadillos, present restricted home ranges, with no migration habits, they may be used for mapping risk areas for infection as well as for understanding the fungus's ecology. Exploring some ecological features, the culture-positive animal was captured in an area of natural vegetation, corresponding to a semideciduous tropical forest, near a watercourse, corroborating the idea that *P. brasiliensis *has great affinity for shade and moist vegetation, near rivers [4,7,14,23]. On the other hand, the molecular detection of *P. brasiliensis *in armadillos from savanna areas indicates the presence of the pathogen in this environment that tends toward an acidic pH and a large quantity of aluminum cations (H+Al) in the soil, which is considered unsuitable for the fungus [7,23]. However, in the present study the amount of aluminum cations (H+Al) in this fragment from the savanna area that provided positive armadillo proved to be quite low, while at one defined site of Cerqueira César, in which one animal was negative, the values of H+Al were relatively high. Obviously, more data are still needed to reach further conclusions on the role of these abiotic factors in the fungal ecology.

In conclusion, studies on *P. brasiliensis *detection in armadillos and other xenarthrans, in connection with the associated ecological factors, represent an apt strategy to obtain fundamental information about the pathogen's ecology as well as the manner in which this fungus interacts with its several hosts, including humans.

## Competing interests

The authors declare that they have no competing interests.

## Authors' contributions

VBRP: Study design, animal necropsy, fieldwork and data collection, laboratory tests, analysis and interpretation of data, writing manuscript. SMGB: Study design, analysis and interpretation of data, writing manuscript. RCT: Fieldwork, analysis and interpretation of data. LB: Geographical location of animals. SCBP: Fieldwork, animal necropsy, analysis and interpretation of data. PSR: Data collection, analysis and interpretation of data. EB: Coordination, study design, fieldwork, analysis and interpretation of data, writing manuscript. All authors read and approved the final manuscript.
